# Comparative genomics reveals candidate carotenoid pathway regulators of ripening watermelon fruit

**DOI:** 10.1186/1471-2164-14-781

**Published:** 2013-11-12

**Authors:** Stefania Grassi, Gabriella Piro, Je Min Lee, Yi Zheng, Zhangjun Fei, Giuseppe Dalessandro, James J Giovannoni, Marcello S Lenucci

**Affiliations:** 1 Dipartimento di Scienze e Tecnologie Biologiche ed Ambientali (Di.S.Te.B.A.), Università del Salento, via Prov.le Lecce-Monteroni,73100 Lecce, Italy; 2 Boyce Thompson Institute for Plant Research, Tower Road, Cornell University campus, Ithaca, New York 14853, USA

**Keywords:** Carotenoid biosynthesis, *Citrullus lanatus*, Fruit ripening, Gene expression, Isoprenoids, Non-climacteric fruits, Transcription factors, Watermelon

## Abstract

**Background:**

Many fruits, including watermelon, are proficient in carotenoid accumulation during ripening. While most genes encoding steps in the carotenoid biosynthetic pathway have been cloned, few transcriptional regulators of these genes have been defined to date. Here we describe the identification of a set of putative carotenoid-related transcription factors resulting from fresh watermelon carotenoid and transcriptome analysis during fruit development and ripening. Our goal is to both clarify the expression profiles of carotenoid pathway genes and to identify candidate regulators and molecular targets for crop improvement.

**Results:**

Total carotenoids progressively increased during fruit ripening up to ~55 μg g^-1^ fw in red-ripe fruits. *Trans*-lycopene was the carotenoid that contributed most to this increase. Many of the genes related to carotenoid metabolism displayed changing expression levels during fruit ripening generating a metabolic flux toward carotenoid synthesis. Constitutive low expression of lycopene cyclase genes resulted in lycopene accumulation. RNA-seq expression profiling of watermelon fruit development yielded a set of transcription factors whose expression was correlated with ripening and carotenoid accumulation. Nineteen putative transcription factor genes from watermelon and homologous to tomato carotenoid-associated genes were identified. Among these, six were differentially expressed in the flesh of both species during fruit development and ripening.

**Conclusions:**

Taken together the data suggest that, while the regulation of a common set of metabolic genes likely influences carotenoid synthesis and accumulation in watermelon and tomato fruits during development and ripening, specific and limiting regulators may differ between climacteric and non-climacteric fruits, possibly related to their differential susceptibility to and use of ethylene during ripening.

## Background

The isoprenoid (also known as terpenoid) pathway is one of the most important and well-studied biosynthetic pathways in plants. It involves cross-talk between the cytosolic mevalonate (MVA) and plastidial 2-*C*-methyl-d-erythritol 4-phosphate (MEP) pathways, to give rise to isopentenyl-diphosphate (IPP), the C5 building block required for the synthesis of a diverse group of natural products that perform numerous biochemical functions in plants. The main branch of the isoprenoid pathway leads to the synthesis and accumulation of carotenoids, C40 terpenoid compounds formed by the condensation of eight isoprene units, within plastids [1]. Carotenoids are important natural pigments found in all plants and algae, in many bacteria and fungi, as well as in some animals. In photosynthetic organisms, carotenoids are always present within chloroplasts associated with the light-harvesting complexes of photosystems, where they gather light energy and transfer it to the chlorophylls, in addition to contributing to protection of the photosynthetic apparatus from photooxidation [2]. Chromoplast synthesized carotenoids accumulate during fruit ripening resulting in dramatic changes in tissue pigmentation. Carotenoids also play an essential role in attracting insects and herbivores that act as floral pollinators and seed dispersion vehicles, including the consumption of plant organs such as ripe fruits as food by humans. When carotenoid-rich foods are ingested, carotenoids are assimilated, metabolized, transported in the plasma by lipoproteins, and stored in various tissues [3] where they display biological activities including acting as antioxidants and free-radical scavengers, reducing the risk of certain types of cancers, and promoting immune responses. In addition, some carotenoids (e.g. β-carotene, β-cryptoxanthin) are precursors of vitamin A, an essential nutrient for humans [4-9]. Carotenoids can undergo multiple structural modifications, namely, cyclization, hydroxylation, and epoxidation, yielding the great variety of carotenoids found in nature comprising more than 600 compounds [10].

Increasing interest is devoted to carotenoid content and composition of food crops because of their important roles in human health [11]. Cultivated watermelon [*Citrullus lanatus* (Thunb.) Matsum. & Nakai var. *lanatus*] is important in the diets of people throughout the world. More than 6% of the world's total area devoted to vegetable production is used for watermelon production [12]. Throughout the Mediterranean basin, watermelon is considered an extremely important agricultural crop, with over 10^6^ tons produced in 2010 at a production value of approximately $3 billion US [12]. The ripening of watermelon fruit is a highly regulated process because color, flavor, aroma, and texture change in a coordinated manner. One of the most noticeable characteristics of watermelon ripening is the dramatic increase in carotenoids. The change in pigmentation is caused by synthesis and accumulation of lycopene within the chromoplasts in watermelon fruit resulting in their characteristic red coloration.

To maximize the health-promoting benefits of carotenoids through increased consumption, characterization of carotenoid synthesis and accumulation in important food crops such as watermelon is essential. Understanding the molecular and genetic components controlling the carotenoid biosynthetic pathway is fundamental for targeted breeding aimed at improving carotenoid-producing watermelon cultivars [13,14].

In the past decade, most carotenoid biosynthesis genes in plants have been identified [15-17]. Identification of the cauliflower Or (Orange) gene further indicates the importance of providing a sink for newly synthesized carotenoids in determining net accumulation [18,19]. Progress in elucidating the mechanisms that control carotenoid biosynthesis and accumulation in plants have been recently achieved using climacteric [e.g. tomato (*Solanum lycopersicum* Mill.)] and non-climateric [pepper (*Capsicum annuum* L.)] fruits as models [20-23], though limited information is available for other species including watermelon.

Different strategies to control carotenoid biosynthesis and accumulation in plant tissues have been reported [20-22]. During flower color development and fruit ripening, transcriptional regulation of carotenoid gene expression has been shown to be a major mechanism by which the biosynthesis and accumulation of specific carotenoids are regulated. Classic examples are found in tomato and pepper (both fruits and flowers), where the synthesis and accumulation of specific carotenoids parallel increased expression of precursor (upstream) carotenogenic genes and reduced expression of downstream (metabolism) genes of the accumulating carotenoids [16,24,25]. The mechanisms of these transcriptional control systems remain poorly understood. Post-transcriptional regulation at the enzymatic level also plays a role in controlling carotenoid biosynthesis and accumulation [26-28]. Metabolic turnover of carotenoids by carotenoid cleavage dioxygenases (CCDs) not only produces important signalling and accessory apocarotenoid molecules, but also helps to maintain the steady-state level of carotenoids in plant tissues. Expression of *CCDs* has been found to negatively regulate carotenoid accumulation [29,30]. A body of evidence has shown that oxidative cleavage of carotenoids is induced under environmental stresses [31,32]. Light and circadian rhythm have been shown to alter the expression of nearly all MEP genes and several carotenoid synthesis genes [33-35] as well as carotenoid catabolism genes [36]. Developmental cues also play important roles in conferring metabolic turnover of carotenoids [28]. In addition, altered plastid biogenesis leading to increased plastid compartment size was associated with elevated chlorophyll and carotenoid levels in *hp* (*high-pigment*) mutants [37-39].

A system analysis approach for transcriptome and metabolic data is presented here to identify putative transcription factors that may impact carotenoid accumulation during watermelon fruit ripening. The pattern of synthesis and accumulation of carotenoids and the expression of carotenoid- and isoprenoid-related genes (specifically, those coding for putative transcription factors) has been analyzed in fresh watermelon during fruit development and ripening in order to clarify the factors influencing accumulation of these bioactive molecules and to identify key regulators and molecular targets for crop improvement.

## Results and discussion

An integrative study combining carotenoid profiles and whole genome transcriptome analysis was performed to gain insight into novel genes associated with and possibly regulating the synthesis and accumulation of carotenoids in watermelon plastids during fruit development and ripening. Analysis of carotenoids in the flesh of watermelon fruits at four successive stages of fruit development and ripening corresponding to the white, white-pink, pink and red-ripe color of the fruit flesh (see methods for details of these stages), are shown in Table 1. Phytoene, phytofluene 1, phytofluene 2, ζ-carotene 2, *cis*-lycopene, *trans*-lycopene, β-carotene, γ-carotene and lutein were identified. Other carotenoids such as ζ-carotene, *cis*-neurosporene, α- and δ-carotene, zeaxanthin and violaxanthin were not detected [LOD (Limit Of Detection) = 0.005 μg g^-1^ fresh weight (fw); LOQ (Limit Of Quantification = 0.015 μg g^-1^ fw) at any of the four stages analyzed.

**Table 1:** Carotenoid content during watermelon fruit ripening

**Carotenoid μg g**^**-1 **^**fw**	**Stages of ripening**			
	**White**	**White-pink**	**Pink**	**Red-ripe**
Phytoene	nd^A^	0.070 ± 0.010^A^	0.410 ± 0.100^B^	0.970 ± 0.100^C^
Phytofluene 1	0.010 ± 0.005^A^	0.040 ± 0.010^A^	0.330 ± 0.100^B^	0.810 ± 0.100^C^
Phytofluene 2	0.020 ± 0.005^A^	0.016 ± 0.005^A^	0.050 ± 0.009^A^	0.070 ± 0.058^A^
ζ-Carotene 2	nd^A^	nd^A^	0.050 ± 0.025^B^	0.130 ± 0.026^C^
*cis*-Lycopene	nd^A^	0.010 ± 0.005^A^	0.060 ± 0.005^B^	0.130 ± 0.030^C^
*trans*-Lycopene	0.030 ± 0.005^A^	3.950 ± 0.750^A^	28.080 ± 4.700^B^	50.540 ± 8.800^C^
β-Carotene	nd^A^	0.010 ± 0.005^A^	0.350 ± 0.220^A^	1.420 ± 0.600^B^
γ-Carotene	nd^A^	0.100 ± 0.019^A^	0.490 ± 0.110^B^	1.160 ± 0.260^C^
Lutein	0.040 ± 0.005^A^	0.020 ± 0.013^A^	0.020 ± 0.012^A^	0.023 ± 0.016^A^
*Car acy:cy*	*1.5*	*32.4*	*34.7*	*20.2*
Total	0.100 ± 0.020^A^	4.216 ± 0.817^A^	29.840 ± 5.281^B^	55.253 ± 9.990^C^

The amount of carotenoids was measured at four stages of fruit ripening: white; white-pink; pink and red-ripe stages. Values are expressed as μg g^-1^ fw. Each value represents the mean result from triplicate ± SD. Data were submitted to one-way analysis of variance (ANOVA), values marked with different capital letters indicate statistically significant difference between ripening stages for a given carotenoid (Holm-Sidak post-hoc test, P < 0.05).

fw, fresh weight; SD, standard deviation; Car, carotenoid; acy, acyclic; cy, cyclic; nd, not detectable. Italic formatting identifies dimentionless ratios.

**Figure 1:**
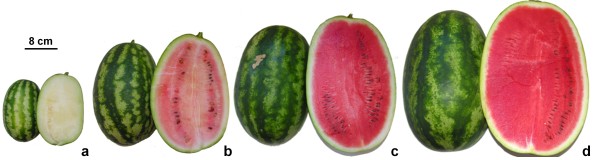
**Fruits of watermelon cultivar Dumara at four different stages of ripening.** Ripening stages were indicated as white **(a)**, white-pink **(b)**, pink **(c)** and red-ripe **(d)**.

The amount of each identified carotenoid varied significantly during fruit ripening (p < 0.001), with the exception of phytofluene 2 and lutein that remained low in all ripening stages. As expected, the total amount of carotenoids (expressed as μg g^-1^ fw) progressively increased during fruit ripening from 0.100 μg g^-1^ fw to 55.253 μg g^-1^ fw at the white and red-ripe stages, respectively. *Trans*-lycopene contributed most to this increase, while the *cis* isomer(s), although increasing in proportion similar to *trans,* represented only 0.2% of the total carotenoid content from the white-pink (where it was detected first) to the red-ripe stage. In terms of color, at the white stage, there is no difference between the inner peel (outer mesocarp) and the placenta [flesh (inner mesocarp and endocarp) tissues] (Figure 1a), while, at the white-pink stage, the flesh tissue started to turn red (Figure 1b) due to lycopene accumulation. *Trans*-lycopene was, in fact, detected at a very low concentration (0.030 μg g^-1^ fw) at the white stage and predominated (3.950 μg g^-1^ fw) at the white-pink stage of ripening. The highest rate of change in accumulation of *trans*-lycopene was observed in the transitional phase between the white-pink and the pink stage where it reached 28.080 μg g^-1^ fw, ~7.1 times higher than that of the previous stage. *Trans*-lycopene increased less than two-fold (50.540 μg g^-1^ fw) by the next (red-ripe) stage. These results confirm our earlier studies, in which lycopene content of the same cultivar ranged between 0 and 47.1 μg g^-1^ fw from the white to the red-ripe stages of ripening [40]. In addition, the amount of lycopene measured at the red-ripe stage concurs and falls within the range (35–112 μg g^-1^ fw) reported for ripe red-fleshed commercial cultivars by Perkins-Veazie et al. [41,42].

At the white stage, lutein, even if present in a very low amount (0.040 μg g^-1^ fw), was the most abundant carotenoid in the watermelon flesh, contributing to 40% of total carotenoids. Phytofluene 1 and 2 were the only acyclic carotenoids detectable at this stage although in amounts close to the LOQ. Starting from the white-pink ripening stage the acyclic carotenoids phytoene and phytofluene 1 increased gradually reaching their maximum (0.970 μg g^-1^ fw, 0.810 μg g^-1^ fw, respectively) in red-ripe fruit. ζ-carotene 2 showed its highest concentration (0.13 μg g^-1^ fw) in fully ripe watermelons but it was detectable only from the pink stage. Phytofluene 2, ζ-carotene 2, *cis*-lycopene and lutein remained at very low levels throughout watermelon fruit development and ripening. In photosynthetic tissues lutein plays a critical role in light-harvesting complex assembly and function, and in photoprotection of photosystems [43]. It is the most abundant carotenoid in the chloroplasts and often accounts for >50% of the total carotenoid pool. The small amounts of lutein found in the non-photosynthetic tissues of the flesh of the young, not yet fully developed, watermelon fruit, could have a role in the protection of the plastid membranes from oxidative stress.

The content of β-carotene was at low levels until the red-ripe stage when β-carotene content was four times higher than the previous stage, representing 2.6% of total carotenoids. At the white-pink ripening stage the amount of γ-carotene was at least 10 times higher than that of β-carotene, but in subsequent stages their contents became similar. Tadmor et al. [44] reported that red watermelons generally have any of the three following carotenoid profiles: 1) high levels of lycopene and small amounts (less than 5% of total carotenoids) of β-carotene; 2) accumulation of mostly lycopene and significant (>10%) β-carotene and 3) lycopene exclusively, with no detectable β-carotene. The first carotenoid pattern seems to best characterize the Dumara cultivar at the red-ripe stage suggesting that mature fruit of this cultivar retain at least some lycopene β-cyclase activity.

**Figure 2:**
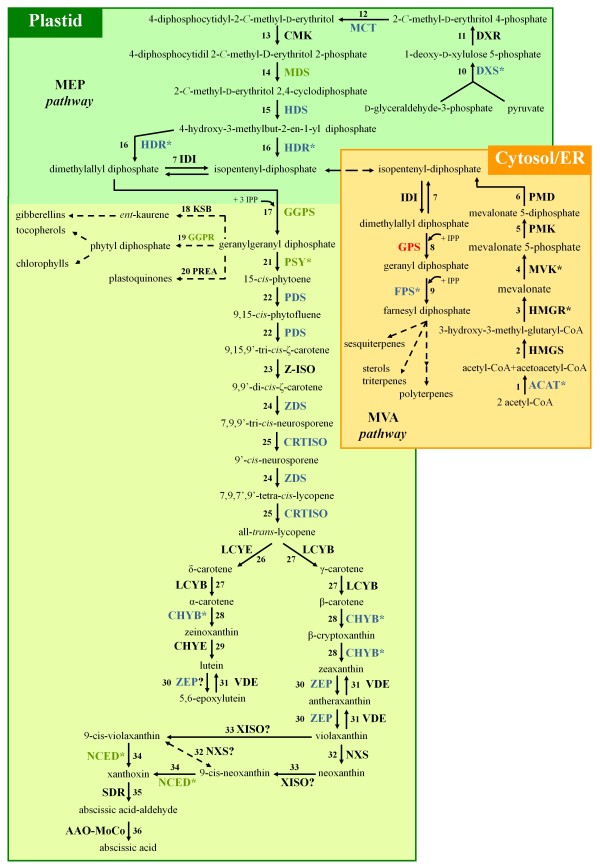
**Summary diagram of pathways related to carotenoid metabolism (MVA, MEP, carotenoid biosynthetic and catabolism pathways).** See below for legend. **1. ACAT**, acetyl-CoA acetyltransferase; **2. HMGS**, 3-hydroxy-3-methylglutaryl-CoA synthase; **3. HMGR**, 3-hydroxy-3-methylglutaryl-CoA reductase; **4. MVK**, mevalonate kinase; **5. PMK**, phosphomevalonate kinase; **6. PMD**, diphosphomevalonate decarboxylase; **7. IDI**, isopentenyl-diphosphate δ-isomerase; **8. GPS**, geranyl-diphosphate synthase; **9. FPS**, farnesyl diphosphate synthase; **10. DXS**, 1-deoxy-d-xylulose-5-phosphate synthase; **11. DXR**, 1-deoxy-d-xylulose-5-phosphate reductoisomerase; **12. MCT**, 2-*C*-methyl-d-erythritol-4-phosphate cytidyltransferase; **13. CMK**, 4-diphosphocytidyl-2-*C*-methyl-d-erythritol kinase; **14. MDS**, 2-*C*-methyl-d-erythritol 2,4-cyclodiphosphate synthase; **15. HDS**, 4-hydroxy-3-methylbut-2-en-1-yl diphosphate synthase; **16. HDR**, 4-hydroxy-3-methylbut-2-enyl diphosphate reductase; **17. GGPS**, geranylgeranyl diphosphate synthase; **18. KSB**, ent-kaur-16-ene synthase; **19. GGPR**, geranylgeranyl diphosphate reductase; **20. PREA**, prenyl transferase; **21. PSY**, phytoene synthase**; 22. PDS**, phytoene desaturase; **23. Z-ISO,** ζ-carotene isomerase; **24. ZDS**, ζ-carotene desaturase; **25. CRTISO**, carotene isomerase; **26. LCYE**, lycopene ϵ-cyclase; **27. LCYB**, lycopene β-cyclase; **28. CHYB**, β-carotene hydroxylase; **29. CHYE**, ϵ-carotene hydroxylase; **30. ZEP**, zeaxanthin epoxidase; **31. VDE**, violaxanthin de-epoxidase; **32. NXS**, neoxanthin synthase; **33. XISO**, xanthophyll isomerase; **34. NCED**, 9-cis-epoxycarotenoid dioxygenase; **35. SDR**, short chain dehydrogenase; **36. AAO-MoCo**, abscisic aldehyde oxidase-molybdenum co-factor. In the diagram, the enzymes with down-regulated gene expression level are indicated in red, the enzymes with up-regulated gene expression level in blue, while the enzymes with gene expression level that increases in the intermediate stages and decreases in the last stage in green. The asterisks identify enzymes encoded by several gene isoforms; the gene expression level of the predominant isoform is shown in the diagram.

The expression profiles of 19,324 genes, among the 23,440 predicted in the watermelon genome assembly [45,46], were generated from trascriptome characterization of watermelon ripening fruits of the Dumara cultivar by Illumina RNA sequencing. Searching the resulting transcriptomics data for genes involved in the pathways related to carotenoid metabolism (MVA, MEP and carotenoid biosynthetic and catabolism pathways) (Figure 2), we identified 40 sequences coding for putative proteins with an e-value lower than 1e-10 and a minimal Reads Per Kilobase of exon model per Million mapped reads (RPKM) value of 8 in at least one of the four ripening stages. This threshold was arbitrarily chosen to filter the sequences with very low counts which may represent estimates of transcript abundance that are too unreliable for inclusion in differential gene expression analysis (see Additional file 1: Table S1 for the expression levels of additional isoprenoid pathway related putative genes for which reads below 8 RPKM, were found). Among the identified sequences, 23 genes were differentially expressed in the flesh tissue during fruit development and ripening (FDR ≤ 0.05) (Table 2).

**Table 2:** **Genes related to mevalonic acid (MVA), 2-C-methyl-d-erythritol-4-phosphate (MEP), carotenoid biosynthetic and catabolic pathways**

	**Description**	**Gene ID**	e-value*	**FDR**
**MVA pathway**	**ACAT2** (Acetyl-CoA acetyltransferase2)	*Cla015696*	4e-172	0.0568
		***Cla010997***	**7e-169**	**0.0050**
	**HMGS** (Hydroxymethylglutaryl-CoA synthase)	*Cla001148*	4e-211	0.0594
	**HMGR1** (3-Hydroxy-3-methylglutaryl-CoA reductase 1)	*Cla001204*	1e-251	0.1373
		*Cla021910*	2e-255	0.1581
	**MVK** (Mevalonate kinase)	*Cla005305*	4e-079	0.2429
		*Cla019880*	9e-112	0.2853
	**PMK** (Phosphomevalonate kinase)	*Cla016251*	2e-019	0.4039
	**PMD** (Diphosphomevalonate decarboxylase)	*Cla020496*	3e-084	0.2037
	**IDI1** (Isopentenyl-diphosphate δ-isomerase1)	*Cla009550*	3e-110	0.0906
	**IDI2** (Isopentenyl-diphosphate δ-isomerase2)	*Cla007929*	1e-113	0.7446
	**GPS** (Geranyl-diphosphate synthase)	***Cla016321***	**2e-016**	**0.0124**
	**FPS1** (Farnesyl diphosphate synthase 1)	*Cla011017*	7e-169	0.0683
		***Cla003330***	**7e-167**	**0.0076**
**MEP pathway**	**DXS** (1-Deoxy-d-xylulose-5-phosphate synthase)	***Cla009346***	**9e-053**	**0.0243**
		***Cla009347***	**8e-153**	**0.0027**
		***Cla009348***	**7e-072**	**0.0017**
		*Cla022299*	2e-220	0.1203
	**DXR** (1-Deoxy-d-xylulose-5-phosphate reductoisomerase)	*Cla019193*	7e-227	0.2373
	**MCT** (2-C-Methyl-d-erythritol 4-phosphate cytidyltransferase)	***Cla004566***	**6e-014**	**0.0036**
	**CMK** ( 4-Diphosphocytidyl-2-C-methyl-d-erythritol kinase)	*Cla011088*	1e-134	0.0718
	**MDS** (2-C-Methyl-d-erythritol 2,4-cyclodiphosphate synthase)	***Cla014654***	**5e-085**	**0.0442**
	**HDS** (4-Hydroxy-3-methylbut-2-en-1-yl diphosphate synthase)	***Cla005033***	**3e-120**	**0.0016**
	**HDR** (4-Hydroxy-3-methylbut-2-enyl diphosphate reductase)	***Cla010297***	**4e-121**	**0.0320**
		***Cla015963***	**2e-149**	**0.0028**
	**GGPS** (Geranylgeranyl diphosphate synthase)	***Cla020121***	**2e-125**	**0.0360**
**Carotenoid biosynthetic pathway**	**PSY** (Phytoene synthase)	***Cla009122***	**2e-228**	**0.0008**
		***Cla005425***	**3e-125**	**0.0324**
	**PDS** (Phytoene desaturase)	***Cla010898***	**1e-174**	**0.0046**
	**CRTISO** (Carotene isomerase)	***Cla017593***	**6e-256**	**0.0264**
	**ZDS** (ζ-carotene desaturase)	***Cla003751***	**3e-224**	**0.0077**
	**LCYB** (Lycopene β-cyclase)	*Cla005011*	1e-207	0.1722
	**LCYE** (Lycopene ϵ-cyclase)	*Cla016840*	2e-214	0.0976
	**CHYB** (β-carotene hydroxylase)	***Cla011420***	**6e-020**	**0.0025**
		***Cla006149***	5e-019	**0.0152**
	**ZEP** (Zeaxanthin epoxidase)	***Cla020214***	**8e-275**	**0.0033**
	**VDE** (Violaxanthin de-epoxidase)	*Cla000667*	7e-102	0.0525
**Carotenoid catabolism**	**NCED1** (9-cis-epoxycarotenoid dioxygenase 1)	***Cla005404***	**1e-249**	**0.0016**
		***Cla009779***	**8e-213**	**0.0187**
	**CCD1** (Carotenoid 9,10(9',10')-cleavage dioxygenase 1)	***Cla015245***	**4e-207**	**0.0030**

Genes were identified through Illumina Sequencing Technology in watermelon fruit using two biological replicas. In bold are indicated the genes differentially expressed during watermelon ripening with a FDR (False Discovery Rate) ≤ 0.05.

*according to Swiss-Prot database.

**Figure 3:**
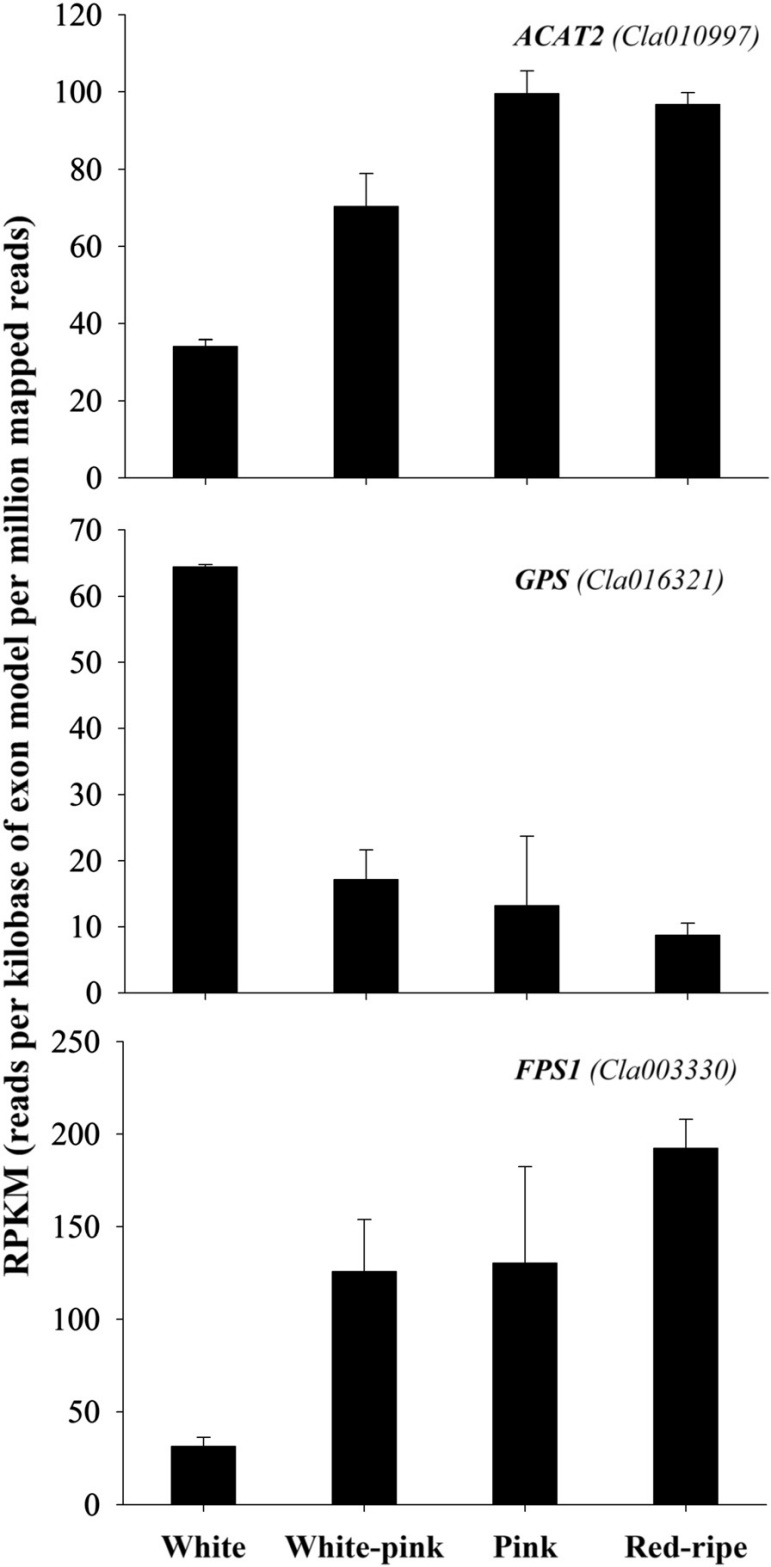
**Expression level of mevalonic acid (MVA) pathway genes during watermelon ripening.** Data were obtained by Illumina RNA Sequencing using two biological replicas and are expressed as Reads Per Kilobase of exon model per Million mapped reads (RPKM). The figure includes only the genes differentially expressed during watermelon fruit ripening with a FDR (False Discovery Rate) ≤ 0.05.

Of the 11 sequences thought to be involved in the upstream MVA pathway leading to isopentenyl-diphosphate (IPP) and dimethylallyl diphosphate (DMAPP) biosynthesis, only one (*Cla010997*) coding for a putative acetyl-CoA acetyltransferase 2 (ACAT2) was differentially expressed during watermelon ripening. Changes in its expression levels in RPKM, are reported in Figure 3.

ACAT2 catalyses the condensation of two acetyl-CoA subunits to form acetoacetyl-CoA thus directing this central metabolite to the MVA pathway. Interestingly ACAT2 mRNA expression was up-regulated approximately three-fold during watermelon ripening suggesting that this enzyme may divert the metabolic flux of acetyl-CoA from the biosynthesis of fatty acids and amino acids toward the synthesis of isoprenoids. 3-Hydroxy-3-methylglutaryl-CoA reductase (HMGR) is a key regulatory enzyme in the pathway and catalyses the formation of MVA from 3-hydroxy-3-methyl-glutaryl-CoA (HMG-CoA). It is a highly regulated enzyme, being subjected to transcriptional, translational, and post-translational control [47]. Although a sequence (*Cla015723*) coding for a putative HMGR1 is differentially expressed in ripening watermelon fruits (FDR = 0.0137) (Additional file 1: Table S1), it was not taken into account in this study having RPKM far below our defined minimal value of 8. However, the very low expression of *hmgr*1 (0–0.3 RPKM) in ripening watermelon fruits supports the assertion that the conversion of HMG-CoA to MVA is the rate limiting step in the biosynthesis of sterols and other isoprenoids [48].

Two different genes (*Cla009550* and *Cla007929*) encoding putative isopentenyl-diphosphate δ-isomerases (IDI1 and IDI2, respectively) were identified. These enzymes convert the relatively un-reactive IPP to the more-reactive electrophile DMAPP. Both genes were stably expressed during watermelon ripening but IDI1 expression was considerably higher than that of IDI2 (708 vs 60 RPKM, as the average value of the four ripening stages, respectively). In *Arabidopsis thaliana* (L.) Heynh. IDI1 and IDI2 are expressed in all organs, with IDI1 less abundant than IDI2. Examination of green fluorescent protein fusions established that IDI1 is mainly located in the plastids, whereas IDI2 is in the mitochondria. Both proteins are also present in the cytosol as a result of their translation from naturally occurring shorter transcripts lacking transit peptides [49].

Three sequences were found to be associated with putative enzymes involved in the IPP down-stream MVA pathway (Table 2). Of these, two (*Cla016321* and *Cla003330*) were differentially expressed during watermelon ripening (Figure 3). The expression of *Cla016321*, coding for a putative geranyl-diphosphate synthase (GPS), was strongly inhibited as soon as the ripening process started. This enzyme is responsible for the conversion of DMAPP in the presence of IPP into geranyl-diphosphate (GPP) which is further converted into farnesyl diphosphate (FPP) in the presence of another molecule of IPP by farnesyl diphosphate synthase (FPS1, the putative protein encoded by *Cla003330*). It is well established that cytosolic IPP, which is then isomerized to DMAPP by the activity of IDIs, contributes to the formation of functional plastidic isoprenoids [50,51]. Down-regulation of *Cla016321* could determine a shift of the MVA produced IPP towards carotenoid biosynthesis. Squalene synthase (encoded in watermelon by *Cla016602*; e-value = 9e-46; FDR = 0.13483; RPKM = 25 ± 7, as the average of the four ripening stages) catalyses the head-to-head condensation of two FPP molecules to form squalene, the first committed step in sterol biosynthesis, and as such was not considered further here.

**Figure 4:**
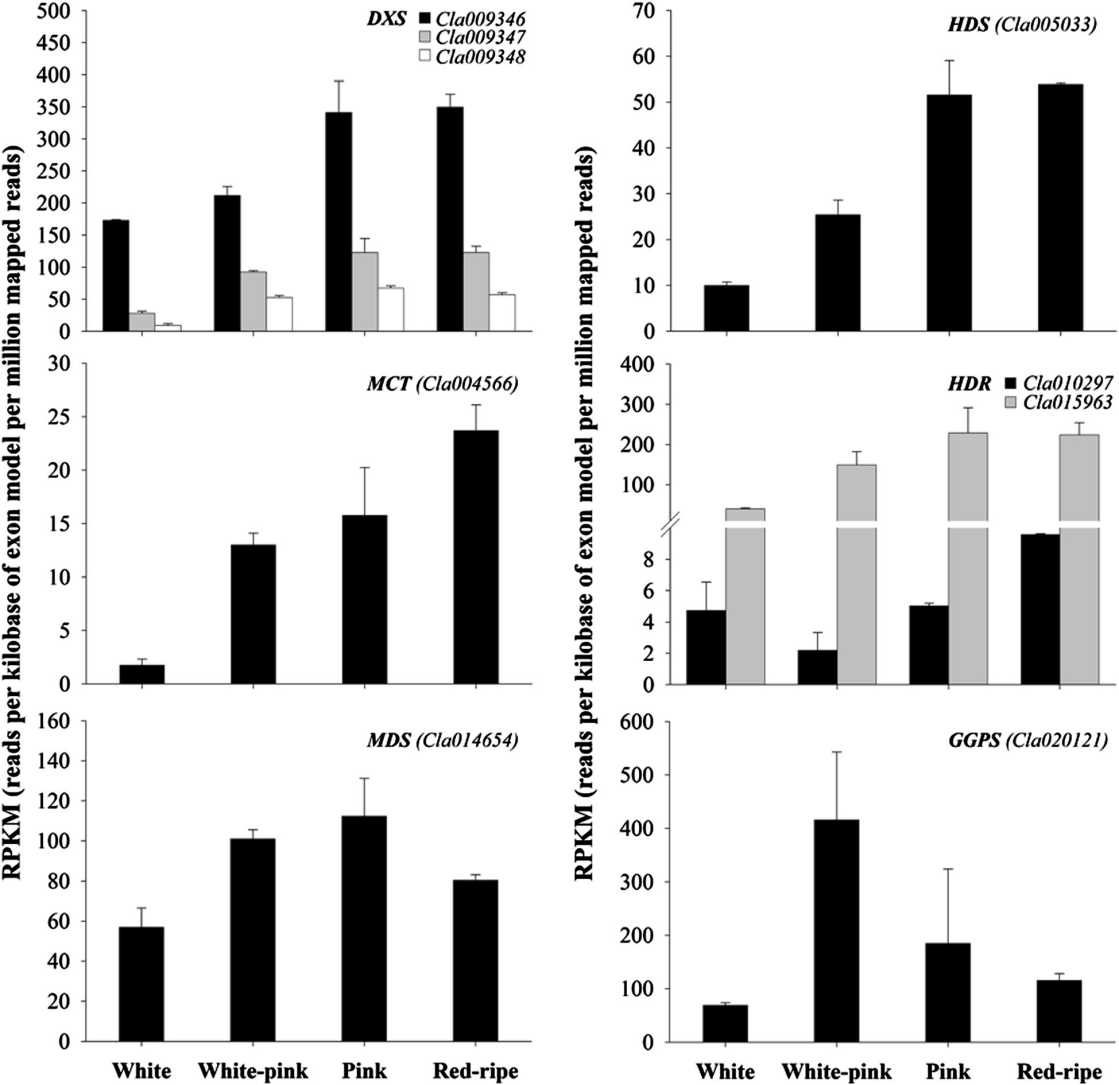
**Expression level of 2-*C*-methyl-d-erythritol-4-phosphate (MEP) pathway genes during watermelon ripening.** Data were obtained by Illumina RNA Sequencing using two biological replicas and are expressed as Reads Per Kilobase of exon model per Million mapped reads (RPKM). The figure includes only the genes differentially expressed during watermelon fruit ripening with a FDR (False Discovery Rate) ≤ 0.05.

In plants, IPP and DMAPP entering in the biosynthesis of carotenoids are mainly synthesized by the MEP pathway in the plastids [1]. In this work 11 sequences were found that are likely to be involved in the up-stream pathway leading to IPP and DMAPP biosynthesis, and 8 were differentially expressed during watermelon fruit development and ripening (Table 2; Figure 4). 1-Deoxy-d-xylulose-5-phosphate synthase (DXS) has been shown to catalyze one of the rate-limiting steps of the MEP pathway [52]. It generates 1-deoxy-d-xylulose-5-phosphate (DXP) by the transketolase-type condensation of pyruvate and d-glyceraldehyde 3-phosphate and is also involved in the biosynthesis of thiamine and pyridoxal (vitamin B_1_ and B_6_, respectively) in bacteria and higher plants [53-55]. Therefore DXS appears to play a key role linking isoprenoid and vitamin biosynthesis. Four genes (*Cla009346*, *Cla009347*, *Cla009348* and *Cla022299*) were found to encode for putative DXS isoforms. Most enzymes of the MEP pathway are encoded by single-copy genes in flowering plants, whereas DXS is typically encoded by a small gene family [35,49,56,57]. The genes of the *DXS* family display differential expression during plant development and in specific organs, suggesting a non-redundant function and possibly a role in production of particular isoprenoids [58,59]. With the exception of *Cla022299*, whose expression did not vary during ripening (FDR = 0.1204; RPKM = 10 ± 3, as the average of the four ripening stages), the other three sequences were differentially expressed (FDR ≤ 0.05) and induced during watermelon fruit ripening (Table 2; Figure 4). This is in agreement with what was found in tomato and pepper fruits [60,61]. In tomato, the highest level of DXS transcripts was detected at the breaker stage, and then decreased during later ripening [62]. DXS mRNA was found to be most abundant in young *Arabidopsis chs5* mutant, maize (*Zea mays* L.) and peppermint (*Mentha × piperita* L.) leaves suggesting that its activity is of critical importance at the early stages of leaf and chloroplast development and confirming its organ and tissue specificity [55,59,63]. The fact that multiple watermelon *DXS* genes are induced during ripening suggests a predominant role of members of this family in driving fruit carotenoid accumulation.

DXP is converted to MEP by the enzyme DXP reductoisomerase (DXR) encoded in watermelon by the gene sequence *Cla019193* whose mRNA expression profile remained stable during fruit ripening (average RPKM = 39 ± 7). MEP is subsequently converted into IPP and DMAPP by the consecutive action of five independent enzymes: 2-*C*-methyl-d-erythritol 4-phosphate cytidyltransferase (MCT), 4-diphosphocytidyl-2-*C*-methyl-d-erythritol kinase (CMK), 2-*C*-methyl-d-erythritol 2,4-cyclodiphosphate synthase (MDS), 4-hydroxy-3-methylbut-2-en-1-yl diphosphate synthase (HDS), and 4-hydroxy-3-methylbut-2-enyl diphosphate reductase (HDR). Isomerization of IPP and DMAPP is catalyzed by the two isomerases IDI1 and IDI2 (encoded by *Cla009550* and *Cla007929*, respectively). The sequences *Cla004566*, *Cla014654*, *Cla005033* and both *Cla010297* and *Cla015963* coding, respectively, for the putative enzymes MCT, MDS, HDS and HDR all showed a significant increase in expression levels during ripening at least up to the pink stage (Figure 4). *Cla011088* encoding for a putative CMK, was stably expressed throughout watermelon fruit ripening (average RPKM = 14 ± 4; FDR = 0.0718).

Geranylgeranyl diphosphate (GGPP), the precursor in the synthesis of all plastid isoprenoids, is generated by geranylgeranyl diphosphate synthase (GGPS) that catalyses the condensation of three IPP and one DMAPP units. The expression of *Cla020121*, encoding for a putative GGPS, increased (approx. six-fold) in the transition between the white and the white-pink stage of ripening, in agreement with the higher rate of synthesis of lycopene between these transitional phases, then progressively decreased at the pink and the ripe-red stages. In other fruits such as mango (*Mangifera indica* L.), GGPS levels were stable throughout the fruit life [64].

**Figure 5:**
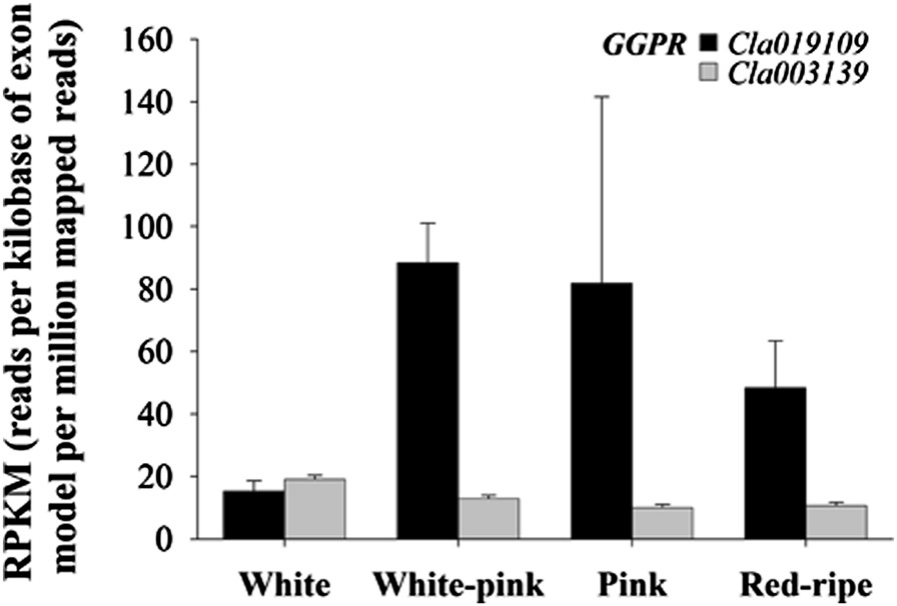
**Expression level of genes related to alternative geranylgeranyl diphosphate (GGPP) catabolism during watermelon ripening.** Data were obtained by Illumina RNA Sequencing using two biological replicas and are expressed as Reads Per Kilobase of exon model per Million mapped reads (RPKM). The figure includes only the genes differentially expressed during watermelon fruit ripening with a FDR (False Discovery Rate) ≤ 0.05.

Since GGPP is a common precursor for the synthesis of phyllochinones, tocopherols, plastoquinones, chlorophylls, gibberellins and carotenoids the expression profiles of genes which may affect carotenoid biosynthesis through competition with phytoene synthase (PSY) for GGPP were also analyzed (Table 3; Figure 5). In watermelon, two sequences (*Cla019109* and *Cla003139*) were found to encode putative geranylgeranyl diphosphate reductases (GGPR) that convert GGPP to phytyl diphosphate in the tocopherol and chlorophyll biosynthetic pathways. Both were differentially regulated during watermelon ripening (Figure 5). While, the expression of *Cla005482* and *Cla005390*, coding for ent-kaur-16-ene synthase (KSB) and prenyl transferase (PREA), diverting the GGPP flux towards the synthesis of gibberellins and plastoquinones, respectively, were stably maintained low during watermelon fruit ripening (average RPKM = 8 ± 2 and 22 ± 5, respectively) presumably to guarantee the synthesis of these fundamental metabolites.

**Table 3:** Genes related to alternative geranylgeranyl diphosphate (GGPP) catabolism

	**Description**	**Gene ID**	e-value*	**FDR**
**Alternative GGPP catabolism**	**GGPR** (Geranylgeranyl diphosphate reductase)	***Cla019109***	**3e-215**	**0.0396**
		***Cla003139***	**3e-146**	**0.0349**
	**KSB** (*ent*-kaur-16-ene synthase)	*Cla005482*	0.0	0.4293
	**PREA** (Prenyl transferase)	*Cla005390*	7e-093	0.2646

Genes were identified through Illumina Sequencing Technology in watermelon fruit using two biological replicas. In bold are indicated the genes differentially expressed during watermelon ripening with a FDR (False Discovery Rate) ≤ 0.05.

*according to Swiss-Prot database.

**Figure 6:**
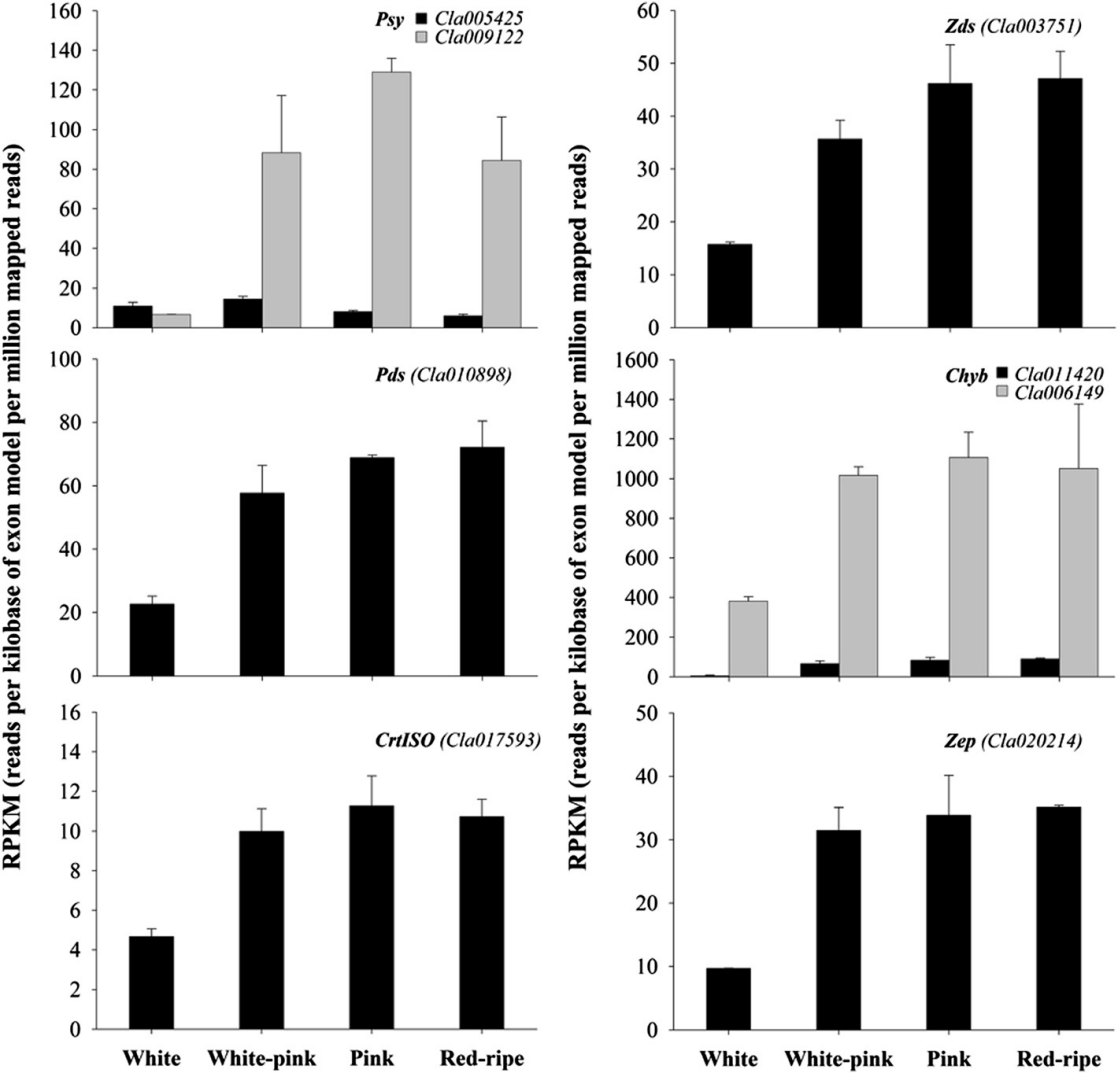
**Expression level of carotenoid metabolism pathway genes during watermelon ripening.** Data were obtained by Illumina RNA Sequencing using two biological replicas and are expressed as Reads Per Kilobase of exon model per Million mapped reads (RPKM). The figure includes only the genes differentially expressed during watermelon fruit ripening with a FDR (False Discovery Rate) ≤ 0.05.

Of the 11 sequences associated with putative enzymes of the carotenoid biosynthetic pathway, 8 were differentially expressed during watermelon ripening (Table 2; Figure 6). As in tomato, two members of the *PSY* gene family were identified and with different expression patterns. *Cla009122* expression level was very low at the white stage but increased sharply as the fruit matured, peaking at the pink and then decreasing at the red-ripe stage. A similar expression profile of a putative *Psy-1* (*WMU38667*) was described by Guo et al. [45] in watermelons of the inbred line 97103. In contrast, *Cla005425* maintained low expression throughout fruit development and ripening, decreasing further in the later stages. The expression profiles are similar to those of tomato *psy-1* and *psy-2*, respectively. *Psy-1* is induced in ripening tomato fruit in association with elevated lycopene accumulation. *Psy-2*, on the other hand, is low in fruit and highly expressed in leaf [65]. Therefore it appears that the PSY encoded by *Cla005425* has a function similar to tomato PSY-2 whereas the product of *Cla009122* is similar to PSY-1.

The expression level of phytoene desaturase (*PDS*; *Cla010898*), carotene *cis-trans* isomerase (*CrtISO*; *Cla017593*) and ζ-carotene desaturase (*Zds*; *Cla003751*) genes increased during fruit development and ripening up to the pink stage and then remained constant (Figure 6). It is well known that PDS catalyzes the desaturation steps, sequentially producing phytofluene and ζ-carotene [66] from phytoene. ZDS with CRTISO are both involved in the steps which sequentially convert 9,9'-di-*cis*-ζ-carotene to pro-lycopene (7,9,7',9'-tetra-*cis*-lycopene) and to all-*trans*-lycopene. Isaacson et al. [67] found that the function of CRTISO paralleled that of the 9,9'-di-*cis*-ζ-carotene desaturase (ZDS) to convert 7,9,9'-tri-*cis*-neurosporene to 9'-*cis*-neurosporene and 7,9,7',9'-tetra-*cis*-lycopene to all-*trans*-lycopene. In tomato, Isaacson et al. [68] reported that deletions in the promoter region and coding region of *CrtISO* resulted in two different color mutants of *tangerine* accumulating pro-lycopene and ζ-carotene instead of all-*trans*-lycopene. This strongly suggests that watermelon *CrtISO* mutations might also cause the salmon yellow or orange mutation that accumulates pro-lycopene and ζ-carotene as major fruit carotenoids [69].

Lycopene β-cyclase (*Lcyb*; *Cla005011*) and lycopene ϵ-cyclase (*Lcye*; *Cla016840*) expression levels were low at the white stage and did not change during watermelon ripening (average RPKM = 23 ± 5 and 6 ± 5, respectively). LCYB is one of the crucial enzymes for carotenoid biosynthesis. LCYB along with LCYE bring about the cyclization of lycopene. Activities of both of these enzymes make α-carotene via δ-carotene, while activity of LCYB alone leads to formation of β-carotene via γ-carotene [70]. In tomato, the down-regulation of this gene may generate a blockade downstream, leading to the accumulation of lycopene in red-ripe fruits [71]. The low expression level of LYCB mRNA that we found in the Dumara cultivar, may permanently maintain low metabolic flux toward cyclic carotenes and xanthophylls during ripening. In contrast to tomato, during watermelon ripening no chloroplast-to-chromoplast transition occurs, rather chromoplasts originate from the differentiation of proplastids. Therefore constant synthesis of β-carotene and lutein, which are present in significant quantities in the purified chloroplasts of unripe tomatoes [72], is not required in watermelon fruits. However, a dramatic reduction in the expression of *Lcyb* and β-carotene hydroxylase gene (*Chyb*), although with differences in the amount of transcript level variation, was recently reported in red-fleshed “ZAOHUA” and pink-fleshed “96B41” watermelon varieties 20–30 days after pollination and related to lycopene accumulation during ripening [73], suggesting that the regulation of *Lcyb* is influenced by watermelon genotype. A gradual decrease of *Lcyb*, which resulted undetectable at the over-ripe stage, was also reported by Guo et al. [45] during watermelon inbred line 97103 ripening.

Two sequences (*Cla011420* and *Cla006149*) coding for putative CHYB isoforms were identified with a similar but quantitatively different expression pattern (Figure 6). The high expression levels of downstream genes *Chyb* and zeaxanthin epoxidase (*Zep*; *Cla020214*), whose expression increased early during watermelon fruit ripening and remained stable over time, may help maintain the amounts of γ- and β-carotene at low levels as intermediate metabolites for other compounds. Similarly the lack of either zeaxanthin and violaxanthin, products of CHYB and ZEP activities, in the watermelon carotenoid profiles at any stage of ripening may be due to their rapid catabolism by dioxygenases [eg. into abscisic acid (ABA)] [73].

**Figure 7:**
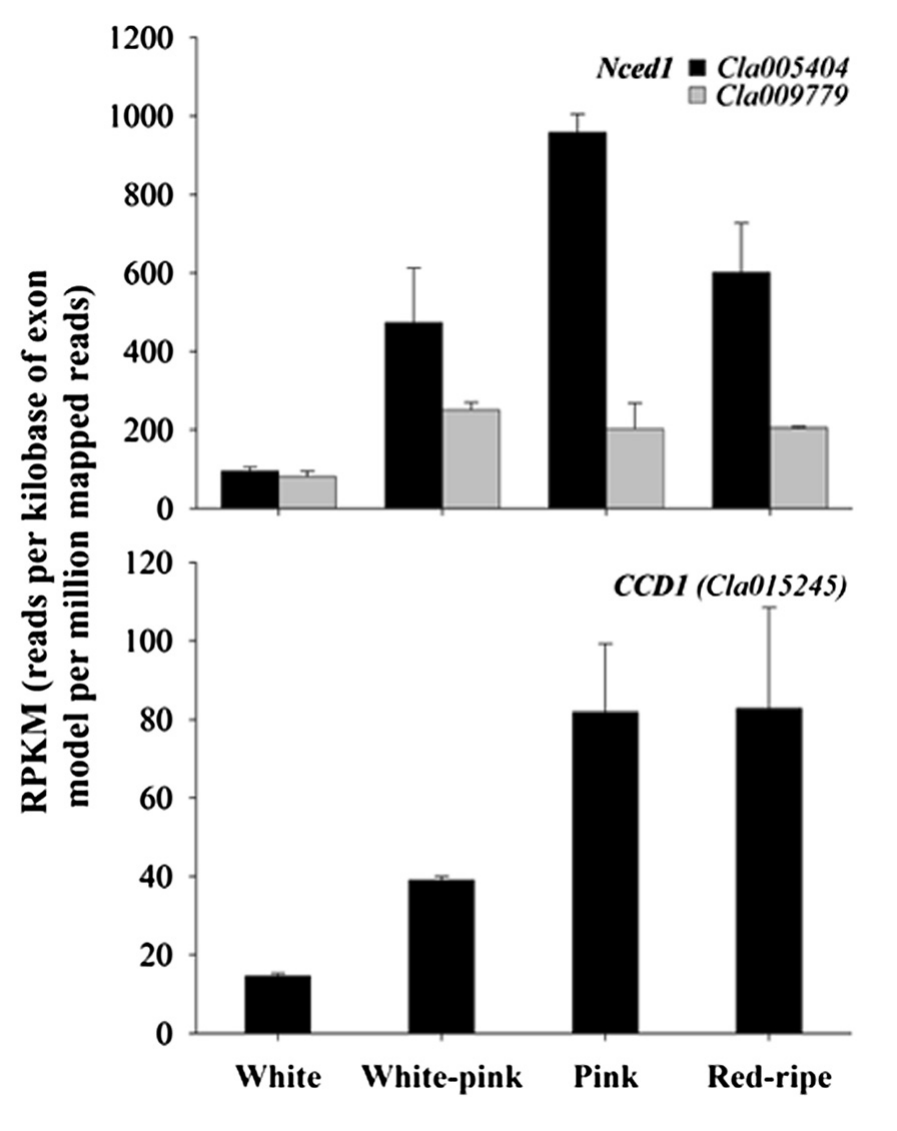
**Expression level of carotenoid catabolism genes during watermelon ripening.** Data were obtained by Illumina RNA Sequencing using two biological replicas and are expressed as Reads Per Kilobase of exon model per Million mapped reads (RPKM). The figure includes only the genes differentially expressed during watermelon fruit ripening with a FDR (False Discovery Rate) ≤ 0.05.

Two members of 9-*cis*-epoxy-carotenoid dioxygenase family, Nced1s (*Cla005404* and *Cla009779*), involved in the cleavage of 9-*cis*-violaxanthin or 9-*cis*-neoxanthin to form C25 epoxy-apocarotenal and xanthoxin (C15), a precursor of ABA [74,75], increased during watermelon ripening with a different expression profile. The transcript *Cla005404* reached the peak of expression at the pink stage whereas the transcript *Cla009779* peaked at the white-pink stage and then slightly declined at the pink and red-ripe stages (Table 2; Figure 7). In addition, one member of *CCD* family [*CCD1* (*Cla015245*)], preferentially involved in volatile compound production from different carotenoid substrates [76,77], was found to increase, reaching the highest values in the pink and red-ripe fruit ripening stages (Figure 7). Now these data suggest that a complex balance between up-regulation and down-regulation of genes belonging to different central pathways of plant cell metabolism generate a flux of metabolic precursors towards lycopene synthesis during watermelon fruit development and ripening. Concurrently, a constitutive low expression level of lycopene cyclase genes creates a blockade downstream, leading to the accumulation of lycopene and demonstrates differential and coordinate regulation of such genes.

In tomato, many regulatory factors operating in the fine control of the isoprenoid pathway have been elucidated [14,22,78]. Many genes (*LeRIN-MADS*, *TAGL1*, *SlERF6*, *AP2*, *MC*, *NAC*-*NOR*, *DET1*, *DDB1*, *CUL4*, *CNR*, *GLK2*, *HB-1*, *U/GLK2* and *TDR4*) have been shown to encode transcriptional regulators affecting the expression of other genes responsible for ripening phenotype, including isoprenoid biosynthesis and accumulation [79-87]. We screened the transcript set of watermelon for similarity against the known mRNA sequences of the above-mentioned tomato transcription factors by BLAST using an e-value threshold of ≤1e − 10. Nineteen sequences encoding putative transcription factors with a RPKM value ≥8 in at least one of the four watermelon ripening stages assayed were identified. Among these, only 6 were differentially expressed in the flesh tissue during fruit development and ripening (FDR ≤ 0.05) (Table 4).

**Table 4:** Putative ripening transcriptional regulator genes

**Description**	**Gene ID**	e-value*	**FDR**
**RIN-MADS** (*S. lycopersicum* MADS-box transcription factor)	*Cla010815*	3e-028	0.1738
	*Cla000691*	2e-026	0.6762
**TAGL1** (*S. lycopersicum* TAGL1 transcription factor)	*Cla009725*	5e-034	0.7333
	*Cla019630*	5e-022	0.6273
**CNR** (*S. lycopersicum* cultivar Ailsa Craig squamosa promoter binding-like protein)	***Cla003384***	**1e-063**	**0.0002**
	***Cla001754***	**2e-043**	**0.0026**
	*Cla013549*	2e-018	0.9119
	***Cla019702***	**6e-16**	**0.0165**
	*Cla009630*	9e-15	0.7472
**NAC-NOR** (*S. lycopersicum* NAC domain protein)	*Cla023408*	4e-017	0.0683
**SIAP2a** (*S. lycopersicum* AP2 transcription factor)	***Cla018268***	**2e-019**	**0.0271**
	*Cla020243*	3e-056	0.3045
	*Cla000701*	6e-081	0.1955
**SlERF6** (*S. lycopersicum* ERF6 mRNA)	*Cla021765*	6e-015	0.0612
	***Cla003789***	**2e-012**	**0.0121**
**DET1** (*S. lycopersicum* deetiolated1 homolog)	*Cla021340*	8e-013	0.2448
**DDB1** (*S. lycopersicum* UV-damaged DNA binding protein 1)	*Cla019536*	0	0.0646
**CUL4** (*S. lycopersicum* cullin 4)	*Cla019583*	1e-151	0.0646
**HB-1** (*S. lycopersicum* homeodomain leucine zipper protein)	***Cla017080***	**3e-019**	**0.0073**

Genes were identified through Illumina Sequencing Technology in watermelon fruit using two biological replicas. In bold are indicated the genes differentially expressed during watermelon ripening with a FDR (False Discovery Rate) ≤ 0.05.

*according to Swiss-Prot database.

Two genes (*Cla010815* and *Cla000691*) were related to the *Solanum lycopersicum* MADS-box transcription factor *RIPENING INHIBITOR* (*LeRIN-MADS*), a member of the *SEPALLATA* (*SEP*) subfamily. Both sequences were constitutively expressed at high levels during watermelon ripening, with average RPKM values of 339 ± 57 (FDR = 0.1738) and 299 ± 24 (FDR = 0.6762), respectively. *MADS-BOX* genes are normally associated with floral development, but RIN-MADS is an essential regulator of tomato fruit ripening. RIN-MADS controls tomato softening and ethylene production by the direct transcriptional regulation of cell-wall-modifying genes and *ACS* (1-aminocyclopropane-1-carboxylate synthase) genes, respectively. In addition, recently, it has been demonstrated to interact with promoters of many genes involved in the major pathways associated with ripening, including carotenoid biosynthesis and accumulation, to both initiate and maintain their expression throughout the ripening process [88-90]. RIN-MADS protein and mRNA are first detected slightly before the breaker stage and maintained throughout ripening [89]. In Ailsa Craig tomato fruits, expression of *LeRIN-MADS* increased 16-fold during ripening [91]. Similarly, expression of a strawberry (*Fragaria x ananassa* Duch.) *LeRIN-MADS* homologous gene, identified by screening of a strawberry fruit cDNA library with a tomato *LeRIN-MADS* cDNA, was enhanced during ripening, suggesting that transcriptional control of ripening is conserved among climacteric and non-climacteric species [79,92,93]. The identification of watermelon *LeRIN-MADS* homolog genes, expressed at high levels throughout the process of fruit ripening, further supports this hypothesis.

Similarly, neither of the two sequences (*Cla009725* and *Cla019630*) homologous to MADS-box transcription factor *TOMATO AGAMOUS-LIKE 1* (*TAGL1*), was differentially expressed during watermelon fruit ripening (FDR = 0.7333 and 0.6273, respectively) (Table 4). The expression of both had average values of 88 ± 10 RPKM and 65 ± 7 RPKM, respectively. In tomato *TAGL1* is induced in the early stages of carpel development and later at the onset of ripening, suggesting it is involved in both processes. Its expression increases during ripening, peaking at the orange stage of fruit development, possibly in relation to ethylene biosynthesis [84,94]. Part of the TAGL1 activity in tomato fruit ripening is, in fact, exerted through regulation of the *ACS2* gene coding for 1-aminocyclopropane-1-carboxylate synthase, the rate-limiting enzyme in ethylene biosynthesis [95]. Ethylene biosynthesis is not essential for watermelon fruit ripening, although varying patterns of ethylene production have been reported in non-climacteric fruits, including watermelons [96] and may explain in part the difference in *TAGL1* expression profiles between tomato and watermelon. The early TAGL1 activity in tomato was shown to be related to expansion of the carpel [84,94]. The large size of watermelon and the constitutive expression of the homologous genes may reflect activity related to the exceptional size of the mature watermelon fruit. In tomato, TAGL1 requires RIN-MADS activity for the induction of lycopene accumulation in ripe fruit. In watermelon, both *RIN* and *TAGL1* are expressed at a substantial level during ripening supporting the idea they have a role in carotenoid synthesis and accumulation.

*TDR4* is another member of the MADS box transcription family, belonging to the SQUAMOSA (SQUA) subfamily, whose expression pattern suggests a possible role during tomato fruit ripening [80] in an ethylene-independent manner [97]. Although three sequences were identified in watermelon with a high similarity to *TDR4* (*Cla010813*, *Cla022037* and *Cla006943* - data not shown), all were expressed at a very low level (below 8 RPKM) and, for this reason, were not considered further. *TDR4*, hence, seems not involved in isoprenoid accumulation during watermelon fruit ripening, but it may influence different biosynthetic pathways in other non-climacteric fruits. A *TDR4* ortholog was, in fact, recently shown to influence anthocyanin biosynthesis during bilberry ripening [98].

**Figure 8:**
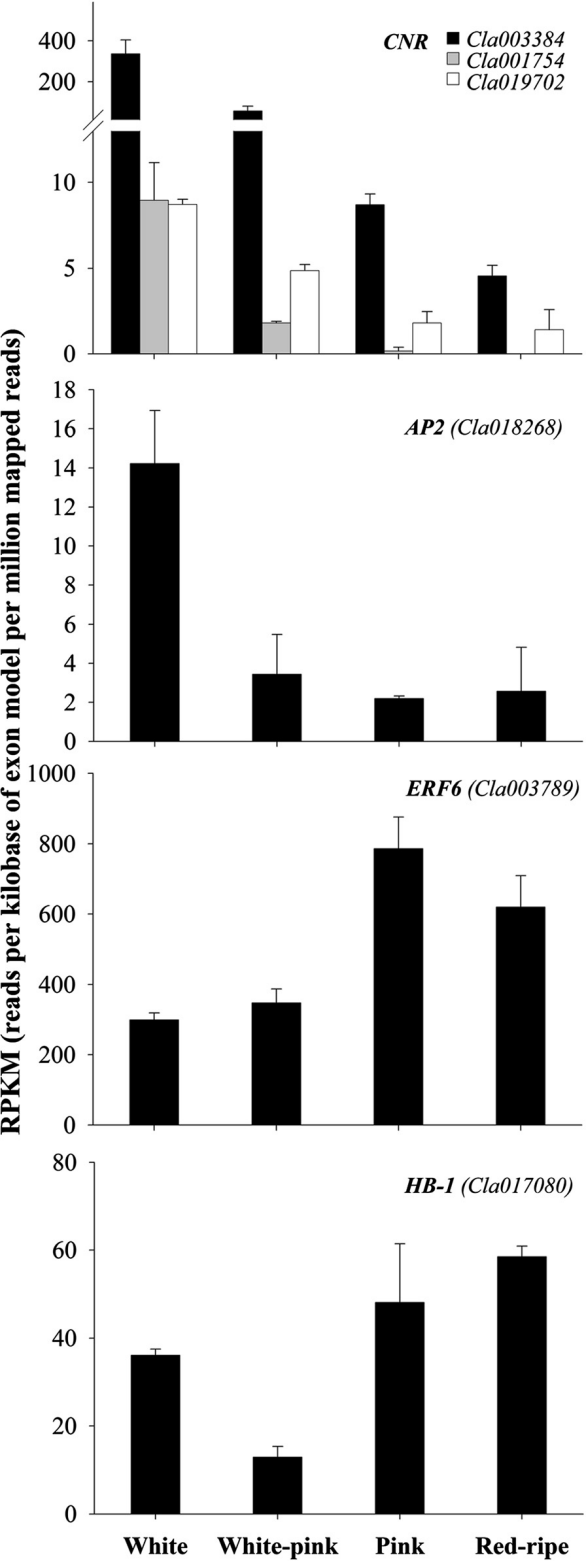
**Expression level of putative ripening transcriptional regulator genes during watermelon ripening.** Data were obtained by Illumina RNA Sequencing and are expressed as Reads Per Kilobase of exon model per Million mapped reads (RPKM). The figure includes only the genes differentially expressed during watermelon fruit ripening with a FDR (False Discovery Rate) ≤ 0.05.

*COLORLESS NON RIPENING* (*CNR*) encodes a transcription factor of the SQUAMOSA promoter-binding protein family (SPB/SPL). It likely controls expression of *SQUA MADS* box genes (such as *TDR4*) by interacting with their promoters. Tomato mutants in this gene show pleiotropic non-ripening phenotypes, including a mealy and pale pericarp [99-101]. Five related sequences were identified in watermelon (Table 4). Two (*Cla013549* and *Cla009630*) exhibited a low and stable expression pattern with average RPKM values of 14.7 ± 1.3 (FDR = 0.9119) and 9.5 ± 1.4 (FDR = 0.7472), respectively. The other three sequences (*Cla003384*, *Cla001754* and *Cla019702*) were differentially expressed during watermelon fruit ripening showing a sharp reduction already in early ripening (Figure 8). In Liberto and Ailsa Craig wild-type tomato fruits *CNR* was transiently expressed at the breaker stage of ripening [82]. CNR is necessary to induce ripening-associated increases in respiration and ethylene synthesis in tomato and other climacteric fruits, in non-climacteric fruits its role remain unclear. The down regulation of the putative *CNR* genes during watermelon ripening suggests it may act as a regulator of isoprenoid accumulation, but with mechanisms different by those operating in climacteric fruits.

Another ripening regulator that pleiotropically controls many aspects of tomato ripening is *NON-RIPENING* (*NAC-NOR*). *Cla023408* showed a high similitude with *NAC-NOR*. In watermelon the expression level of *Cla023408* did not significantly change during fruit ripening (average RPKM = 27 ± 11) suggesting that NAC-NOR protein is not limiting in watermelon fruit ripening as it is in tomato [91].

In tomato *APETALA2a* (*SlAP2a*) transcription factor, a member of the *APETALA2/ETHYLENE RESPONSE FACTOR* (*AP2/ERF*) superfamily, influences fruit ripening via regulation of ethylene biosynthesis and signaling [86,102]. In tomato, RIN-MADS, NAC-NOR and CNR positively regulate *SIAP2a* expression which is, in turn, a negative regulator of ripening and ethylene production. *SIAP2a* is expressed at a relatively low level in flowers and early fruit stages but it is strongly up-regulated between the mature green and breaker stages and is highly expressed to the red-ripe stage [86,102]. Three homologs (*Cla018268*, *Cla020243* and *Cla000701*) of *SIAP2a* were identified in the watermelon transcriptome. *Cla018268* was expressed at a relatively low level and down-regulated during ripening (Figure 8). On the contrary, *Cla020243* and *Cla000701* expression was almost constant during ripening although with differences in their relative expression levels. *Cla020243* was in fact expressed at a relatively low level (average RPKM = 22 ± 4; FDR = 0.3045) whereas *Cla000701* was highly expressed throughout ripening (average RPKM = 294 ± 78; FDR = 0.1955) suggesting *Cla000701* is the most likely functional ortholog of *SlAP2a* in watermelon, though its role in non-climacteric ripening may be in a different context than through regulation of ethylene response.

An additional member of the AP2/ERF superfamily, *SlERF6*, has been recently identified to play an important role in tomato fruit ripening and carotenoid accumulation acting as a negative regulator of two of the predominant nutritional compounds of tomato, lycopene and β-carotene [23]. Ethylene response factors (ERFs) are plant transcriptional regulators mediating ethylene-dependent gene expression via binding to the GCC motif found in the promoter region of ethylene-regulated genes. Two watermelon genes (*Cla021765* and *Cla003789*) have high similarity with *SlERF6*. While *Cla021765* was constitutively expressed during ripening with no significant changes over time (average RPKM = 42 ± 12), *Cla003789* expression was up regulated during the late stages of watermelon ripening, peaking at the pink stage (Figure 8). It has been suggested that SlERF6 may integrate ethylene-dependent and independent regulatory activities to allow for the fine-tuning of signal outputs.

Putative homologs of tomato components of the light signal transduction pathway, such as *DE-ETIOLATED1* (*DET1*; originally identified as *HIGH PIGMENT* [*hp2*]), *UV-DAMAGED DNA BINDING PROTEIN1* (*DDB1*; originally *hp1*) and *CULLIN-4* (*CUL4*) were identified in watermelon (sequences *Cla021340*, *Cla019536* and *Cla019583*, respectively – Table 4). All three genes were expressed at a low level during watermelon fruit ripening with no significant variation (FDR > 0.05) suggesting they are probably not rate limiting in isoprenoid biosynthesis and associated fruit pigmentation.

*GLK2 (Golden 2-like*) transcription factor (of the GARP family Myb transcription factor) determines chlorophyll accumulation and distribution in developing tomato fruits by controlling chloroplast formation. In tomato it is expressed in fruits where it accumulates at the mature-green stage [87]. In watermelon fruits the sequence *Cla003729* was identified as putative homolog of *GLK2* (e-value = 3e-11) but no transcript reads were generated by the Illumina RNA sequencing technology suggesting it is not expressed during ripening. This is in accordance with the evidences that watermelon flesh chromoplasts do not de-differentiate from chloroplasts as occurs in tomatoes but, mainly, from undifferentiated proplastids [103].

Another transcription factor, the homeobox protein *LeHB-1*, was reported to regulate fruit ripening through transcriptional control of *1-aminocyclopropane carboxylic acid oxidase 1* (*ACO1*) expression [83]. *LeHB-1* is highly expressed in tomato flower buds, senescing flowers, and developing immature and mature green fruits, but its mRNA declined during ripening and is maintained at a stable but relatively low level in red-ripe fruits [83]. *Cla017080* the putative watermelon homolog of *LeHB-1* was expressed in developing watermelon fruits at the white stage, down regulated at the beginning of carotenoid accumulation (white-pink stage) and up-regulated at the pink and red-ripe stages of ripening (Figure 8). Ethylene is not essential for non-climacteric fruits, however, recent studies indicate that ethylene and/or a modulated sensitivity to ethylene might participate in physiological changes during non-climacteric fruit development [104-107]. Indeed, many non-climacteric fruits, including watermelon, are highly sensitive to exogenous ethylene [108,109]. In addition *Cla017080* may regulate isoprenoid accumulation in an ethylene independent way by controlling other regulative factors. Putative *LeHB-1* sites have been, in fact, found in the promoters of a number of ripening related genes, including *LeRIN-MADS* and *NAC-NOR*.

## Conclusions

Taken together, these results suggest maintenance of many regulators in the watermelon genome in common with tomato, yet suggest a complex and, for many aspects, different regulatory system for isoprenoid biosynthesis between these fruits. While a common set of metabolic and regulatory genes influences carotenoid accumulation during development and ripening, specific regulatory systems may also differ possibly related to the different ripening physiologies of climacteric and non-climacteric fruits. As such, these data represent the starting point for characterizing conserved and distinct regulatory functions of isoprenoid biosynthesis in climacteric and non-climacteric species. They also provide information and targets for plant researchers and breeders to test as potential tools for watermelon crop improvement. Since this study was done only at the transcriptional level, subsequent experimentation is required to see if other changes arise at other levels of cellular regulation.

## Methods

### Plant cultivation

Watermelon (*Citrullus lanatus* (Thunb.) Matsum. & Nakai var. *lanatus*) cultivar Dumara was used in the present study. Dumara is a twenty year old cultivar but it is still one of the most important commercial selections worldwide and produces elongated fruits characterized by green skin with dark green stripes and sweet, firm flesh containing seeds. Sowing was carried out on 19 February 2010 in plug-seedling trays. One-month-old watermelon seedlings were transplanted at a spacing of approximately 200 cm and 250 cm between rows into a sandy soil of an open-field in the province of Lecce in southern Italy (latitude 40°23’16”80 N, longitude 17°57’41”40E; decimal degrees 40.3881; 17.9615). After transplanting, drip irrigation was applied with 4 L h^-1^, for 1–3 h, at 1–2-day intervals, as determined by potential evapotranspiration at the research station, climate data and crop coefficients as defined by FAO [110]. Drippers were placed at 0.4 m intervals along the irrigation line. Chemical fertilizer solution (145 kg N ha^-1^, 140 kg P_2_O_5_ ha^-1^, 210 kg K_2_O ha^-1^) was added to water irrigation by pump injection twice a week. The production methods also included hand-weeding and plant-pathogen control with synthetic chemical pesticides. Imidacloprid (200 g L^-1^) was used to reduce aphids, acetamiprid (200 g L^-1^) was applied to reduce thrips and abamectine (18 g L^-1^) was used to reduce mites.

### Fruit sampling

Watermelon fruits were harvested from the rows at different ripening stages. Three independent samples of at least 3 injury-free watermelon fruits were hand harvested randomly at four ripening stages indicated as white [~10 days after pollination (DAP)]: small fruit size (approx. 18 cm long × 10 cm wide) and white flesh; white-pink (~18 DAP): not yet mature medium sized fruit (approx. 31×18 cm) with white-pink flesh; pink (~28 DAP): large fruit size (approx. 35×23 cm) with pink flesh and green tendril; red-ripe (~34 DAP): fully expanded (approx. 40×28 cm) mature fruit with red flesh, brown tendril and yellow ground spot (Figure 1). Watermelon fruits were quickly delivered to the laboratory and cut longitudinally from the stem-end to the blossom-end through the ground spot.

The soluble solid content (°Brix) was measured immediately by cutting a wedge of flesh from the heart area (between locules and the fruit centre) and squeezing the juice into a digital refractometer (Atago PR-100, NSG Precision Cells, Inc, Farming dale, NY, US) calibrated with a 10% sucrose solution. Since soluble solid content increases during watermelon ripening, the measured values were used to identify the four ripening stages as follows: white stage (2-3°Brix), white-pink stage (4-5°Brix), pink stage (7-8°Brix) and red-ripe stage (10-12°Brix).

For all further analyses, flesh samples were taken from the heart area of each watermelon. These tissues were immediately frozen in liquid nitrogen and stored at −80°C until use.

### Carotenoid extraction and HPLC analysis

Frozen flesh samples from each fruit stage were rapidly homogenized with a laboratory blender (Waring Laboratory and Science, Torrington, CT, US). Carotenoid extraction and determination were conducted as described by Alba et al. [111]. Frozen homogenates (300 mg) were subjected to extraction of carotenoids with 300 mL of tetrahydrofuran and 50 μL of Mg carbonate (0.3 g/mL). The samples were homogenized in a FastPrep machine (FastPrep FP120, Qbiogene, Inc., Carlsbad, CA, US) and resulting homogenates were filtered with a Spin-X filter (Corning International K.K., Tokyo, Japan). The samples were re-extracted with 300 μL of 5% w/v butylated hydroxytoluene in methanol.

Carotenoids were partitioned into 375 μL of petroleum ether using 150 mL of 25% NaCl. The extract was evaporated to near dryness using a Vacufuge 5301 Centrifugal Vacuum Concentrator (Krackeler Scientific Inc., Albany, NY, US), suspended in 500 μL di methyl *t*-butyl ether and 475 μL di methanol and passed through a syringe filter (GE Osmonics, Minnetonka, MN, US) prior to injection onto a C30 carotenoid column (Waters, Milford, MA, US).

HPLC employed a Summit HPLC system and a PDA-100 photodiode array detector (Dionex, Sunnyvale, CA, US). The elution gradient consisted of 5 min at 100% methanol, a 20-min ramp to 95% *t*-butyl ether, 5 min at 95% *t*-butyl ether, and a 5-min ramp returning the system to 100% methanol. The column was equilibrated with 100% methanol for 10 min before each run. Spectra were collected at 348, 434, 450 and 471 nm and pigments were identified via co-migration with purified standards and/or by their pigment-specific absorbance spectra. Results are presented as mean value ± standard deviation of at least three independent replicated experiments (n = 3). Statistical analysis was based on a one-way ANOVA test. The post-hoc method by Holm-Sidak was applied to establish significant differences between means with a confidence level of 95%. All statistical comparisons were performed using the SigmaStat Version 3.11 software (Systat Software Inc., Chicago, IL, US).

### RNA-Seq experiment

#### Total RNA isolation

Total RNA was isolated from frozen flesh homogenates from every fruit stage using the RNeasy Plant Mini kit (Qiagen, Hilden, Germany). RNA quality and quantity were determined using a NanoDrop spectrophotometer and denaturing agarose gel electrophoresis [112]. Only RNAs with an OD260:OD280 ratio >1.80 and no discernible degradation were used for preparing samples for sequencing of mRNA.

#### Preparation of cDNA libraries and sequencing (RNA-seq)

Sample preparation and multiplex sequencing was essentially as described in Zhong et al. [113]. In summary, samples for sequencing of mRNA were prepared using mRNA-Seq Sample Prep Kit (Illumina, San Diego, CA, US) following manufacturer’s instructions. PolyA^+^RNA was extracted from 10 μg of each total RNA sample using poly-T oligo-attached magnetic beads. The mRNA was eluted in 10 mM Tris–HCl and fragmentated in small pieces using divalent cations under elevated temperature. For the first strand of cDNA synthesis, cleaved mRNA fragments were mixed with random primers, incubated at 70°C for 5 minutes, and then transferred to an ice bath. 5× First strand buffer, 100 mM DTT, 25 mM dNTP mix and RNase OUT were added to the previous mix obtaining a total volume of 19 μl; this reaction mix was incubated for 2 minutes at 25°C. Then, SuperScript II (Invitrogen, Carlsbad, CA, US) was added to the sample that was incubated at 25°C for 10 minutes, 42°C for 50 minutes, 70°C for 15 minutes. The resulting first strand cDNA was used to make second strand cDNA in a reaction mix containing GEX Second strand buffer, 25 mM dNTPs, DNA polymerase I, RNase H in a total volume of 100 μl; this reaction mix was incubated for 2.5 hours at 16°C. The resulting double stranded cDNA was then purified using the QIAquick PCR purification kit (Qiagen), following the manufacturer’s instructions. The cDNA was blunt ended with End Repair Enzyme (NEB) in the presence of 2.5 mM dNTPs (NEB) and 10 mM ATP. Adenine nucleotide was ed to the 3' ends of the blunt ended cDNA with Klenow DNA Polymerase (3' to 5' exominus) in the presence of 1 mM dATP (NEB) by incubating at 37°C for 30 minutes. The end labeled double stranded cDNA was purified with a MinElute PCR purification kit (Qiagen). The double stranded cDNA with A-nucleotides on 3' ends was ligated with adapters (Illumina) using T4 DNA ligase at room temperature for 15 minutes. The samples were then purified with MinElute PCR purification kit (Qiagen). The products of the ligation reaction were purified on 2% agarose gel selecting 200 bp (±25 bp) templates. Subsequently, the cDNA was amplified with two adapter primers (Illumina) with initial denaturing step at 98°C for 30 seconds, followed by 15 cycles at 98°C for 10 seconds, 65°C for 30 seconds, 72°C for 30 seconds with a final extension cycle at 72°C for 5 minutes. The PCR product was purified with Qiaquick PCR purification kit. DNA size, purity and concentration were checked by an Agilent 2100 bioanalyzer (Agilent Technologies, Santa Clara, CA, US). Libraries were barcoded and multiplexed in collections of four samples per lane of sequencing. Sequencing was performed on an Illumina GAII at the Cornell Weill Medical School campus in New York City. A total of 5.7-10.7 million reads were obtained for each library. Raw RNA-seq reads have been deposited into the NCBI sequence read archive (SRA) under accession SRA102510.

#### Gene expression analysis of RNA-Seq data

RNA-Seq reads were first aligned to ribosomal RNA (rRNA) sequence database [114] using Bowtie allowing up to two mismatches [115], to remove any possible rRNA contaminations. The resulting filtered reads were aligned to the watermelon reference genome [116] using TopHat [117] allowing one segment mismatch. Following alignments, raw counts for each watermelon gene were normalized to Reads Per Kilobase of exon model per Million mapped reads (RPKM). Two biological replicas from distinct watermelon fruits (n = 2) were performed.

To identify differentially expressed genes during watermelon fruit development, the RNA-seq expression data were first transformed using the getVarianceStabilizedData function in the DESeq package [118]. The variance-stabilizing transformed RNA-Seq expression data were then fed to the LIMMA package, and F tests were performed [119]. Raw p-values of multiple tests were corrected using FDR [120]. Genes with FDRs less than 0.05 were identified as differentially expressed genes.

## Abbreviations

ABA: Abscisic acid; ACAT: Acetyl-CoA acetyltransferase; ACO: 1-aminocyclopropane carboxylic acid oxidase; ACS: 1-aminocyclopropane-1-carboxylate synthase; ANOVA: Analysis of variance; CCDs: Carotenoid cleavage dioxygenases; CHYB: β-carotene hydroxylase; CMK: 4-diphosphocytidyl-2-*C*-methyl-d-erythritol kinase; DAP: Day after pollination; CRTISO: Carotene *cis-trans* isomerase; DMAPP: Dimethylallyl diphosphate; DXP: 1-deoxy-d-xylulose-5-phosphate; DXR: 1-deoxy-d-xylulose-5-phosphate reductoisomerase; DXS: 1-deoxy-d-xylulose-5-phosphate synthase; ERFs: Ethylene response factors; FPS: Farnesyl diphosphate synthase; FDR: False discovery rate; FPP: Farnesyl diphosphate; GGPP: Geranylgeranyl diphosphate; GGPR: Geranylgeranyl diphosphate reductase; GGPS: geranylgeranyl diphosphate synthase; GPP: Geranyl-diphosphate; GPS: Geranyl-diphosphate synthase; HDR: 4-hydroxy-3-methylbut-2-enyl diphosphate reductase; HDS: 4-hydroxy-3-methylbut-2-en-1-yl diphosphate synthase; HMG-CoA: 3-hydroxy-3-methyl-glutaryl-CoA; HMGR: 3-hydroxy-3-methylglutaryl-CoA reductase; hp: High-pigment; IDI: Isopentenyl-diphosphate δ-isomerase; IPP: Isopentenyl-diphosphate; KSB: Ent-kaur-16-ene synthase; LCYB: Lycopene β-cyclase; LCYE: Lycopene ϵ-cyclase; LOD: Limit of detection; LOQ: Limit of quantification; MCT: 2-*C*-methyl-d-erythritol 4-phosphate cytidyltransferase; MDS: 2-*C*-methyl-d-erythritol 2,4-cyclodiphosphate synthase; MEP: 2-*C*-methyl-d-erythritol 4-phosphate; MVA: Mevalonate; PDS: Phytoene desaturase; PREA: Prenyl transferase; PSY: Phytoene synthase; RPKM: Reads per kilobase of exon model per million mapped reads; ZDS: ζ-carotene desaturase; ZEP: Zeaxanthin epoxidase.

## Competing interests

The authors declare that they have no competing interests.

## Authors’ contributions

SG carried out most of the biochemical and molecular studies and helped to draft the manuscript, GP gave a substantial contributions to the conception and design of the experimental work, JML contributed to the RNA-sequencing experiments, YZ and ZF substantially contributed to the gene expression analysis of RNA-sequencing data and performed most of the statistical analysis, GD and JJG made substantial contributions to the interpretation of data, revised critically the manuscript for important intellectual content, and gave the final approval of the version to be published, MSL conceived the study, participated in its design and coordination, carried out some of the biochemical studies and wrote the manuscript. All authors read and approved the final manuscript.

## Supplementary Material

Additional file 1: Table S1 Expression levels of additional putative genes related to the isoprenoid pathway for which no reads (or reads below 8 RPKM) were found.Genes were identified through Illumina Sequencing Technology in watermelon fruit using two biological replicas. In bold are indicated the genes differentially expressed during watermelon ripening with a FDR (False Discovery Rate) ≤ 0.05. Click here for file
