# Supplementary material for: Comparative genomics reveals candidate carotenoid pathway regulators of ripening watermelon fruit
Source: BMC Genomics. 2013 Nov 12;14:781. doi: 10.1186/1471-2164-14-781 (PMC3840736; doi:10.1186/1471-2164-14-781)
Supplement: Additional file 1: Table S1 — Expression levels of additional putative genes related to the isoprenoid pathway for which no reads (or reads below 8 RPKM) were found.Genes were identified through Illumina Sequencing Technology in watermelon fruit using two biological replicas. In bold are indicated the genes differentially expressed during watermelon ripening with a FDR (False Discovery Rate) ≤ 0.05. [file 1471-2164-14-781-S1.docx]

**Additional file 1 TableS1. Expression levels of additional putative genes related to the isoprenoid pathway** **for which no reads (or reads below 8 RPKM) were found**.

| **Description** | **Gene ID** | **e-value*** | **FDR** | **Expression level during ripening**  **(RPKM)** | | | |
| --- | --- | --- | --- | --- | --- | --- | --- |
|  |  |  |  | **White** | **White-pink** | **Pink** | **Red-ripe** |
| **HMGR1** (3-Hydroxy-3-methylglutaryl-CoA reductase 1) | ***Cla015723*** | **3e-257** | **0.0137** | **0.13** | **0.14** | **0.00** | **0.34** |
| **PMK** (Phosphomevalonate kinase) | *Cla008842* | 7e-018 | 0.1059 | 3.30 | 2.05 | 3.70 | 5.12 |
| **DXS** (1-Deoxy-d-xylulose-5-phosphate synthase) | ***Cla000871*** | **4e-303** | **0.0170** | **0.83** | **5.04** | **0.83** | **1.34** |
|  | *Cla000872* | 4e-154 | 0.0866 | 0.12 | 0.27 | 0.00 | 0.24 |
|  | *Cla018798* | 3e-272 | 0.1686 | 5.57 | 7.10 | 4.74 | 3.09 |
| **GGPS** (Geranylgeranyl diphosphate synthase) | *Cla008507* | 5e-115 | 0.2846 | 0.70 | 0.16 | 0.21 | 0.56 |
| **PSY** (Phytoene synthase) | *Cla003169* | 3e-123 | 0.1028 | 1.00 | 0.07 | 0.48 | 0.40 |
| **PDS** (Phytoene desaturase) | *Cla020261* | 1e-200 | 0.5097 | 5.69 | 3.67 | 4.21 | 4.05 |
| **CRTISO** (Carotene isomerase) | *Cla011810* | 3e-016 | 0.3888 | 1.89 | 3.65 | 2.44 | 2.91 |
| **ZEP** (Zeaxanthin epoxidase) | *Cla002818* | 9e-014 | 0.1469 | 2.99 | 5.63 | 6.47 | 5.66 |
| **NCED1** (9-cis-epoxycarotenoid dioxygenase 1) | *Cla019578* | 6e-209 | 0.2081 | 0.63 | 0.05 | 0.13 | 0.07 |
|  | *Cla002942* | 9e-179 | 0.9313 | 0.20 | 0.04 | 0.05 | 0.10 |

Genes were identified through Illumina Sequencing Technology in watermelon fruit using two biological replicas. In bold are indicated the genes differentially expressed during watermelon ripening with a FDR (False Discovery Rate) ≤ 0.05.

* according to Swiss-Prot database.
